# Real-time compression feedback for patients with in-hospital cardiac arrest: a multi-center randomized controlled clinical trial

**DOI:** 10.1186/s40560-019-0357-5

**Published:** 2019-01-22

**Authors:** Reza Goharani, Amir Vahedian-Azimi, Behrooz Farzanegan, Farshid R. Bashar, Mohammadreza Hajiesmaeili, Seyedpouzhia Shojaei, Seyed J. Madani, Keivan Gohari-Moghaddam, Sevak Hatamian, Seyed M. M. Mosavinasab, Masoum Khoshfetrat, Mohammad A. Khabiri Khatir, Andrew C. Miller, for the MORZAK Collaborative

**Affiliations:** 1 Anesthesiology Research Center, Anesthesia and Critical Care Department, Loghman Hakim Hospital, Shahid Beheshti University of Medical Sciences, Tehran, Iran; 2 Trauma Research Center, Nursing Faculty, Baqiyatallah University of Medical Sciences, Tehran, Iran; 3 Tracheal Diseases Research Center, Anesthesia and Critical Care Department, Masih Daneshvari Hospital, Shahid Beheshti University of Medical Sciences, Tehran, Iran; 4 Anesthesia and Critical Care Department, Hamedan University of Medical Sciences, Hamedan, Iran; 5 Medicine Faculty, Trauma Research Center, Baqiyatallah University of Medical Sciences, Tehran, Iran; 6 Department of Internal Medicine, Shariati Hospital, Tehran University of Medical Sciences, Tehran, Iran; 7 Anesthesia and Critical Care Department, Alborz University of Medical Sciences, Karaj, Iran; 8 Anesthesiology Research Center, Anesthesia Care Department, Modares Hospital, Shahid Beheshti University of Medical Sciences, Tehran, Iran; 9 Anesthesiology Research Center, Anesthesia and Critical Care Department, Khatam-o-anbia Hospital, Zahedan University of Medical Sciences, Zahedan, Iran; 10 Anesthesiology Research Center, Anesthesia and Critical Care Department, Taleghani Hospital, Shahid Beheshti University of Medical Sciences, Tehran, Iran; 11 Department of Emergency Medicine, Vident Medical Center, East Carolina University Brody School of Medicine, 600 Moye Blvd, Greenville, NC 27834 USA

**Keywords:** CPR, Resuscitation, Chest compression, Cardio First Angel™, Critical care, Intensive care

## Abstract

**Objective:**

To determine if real-time compression feedback using a non-automated hand-held device improves patient outcomes from in-hospital cardiac arrest (IHCA).

**Methods:**

We conducted a prospective, randomized, controlled, parallel study (no crossover) of patients with IHCA in the mixed medical–surgical intensive care units (ICUs) of eight academic hospitals. Patients received either standard manual chest compressions or compressions performed with real-time feedback using the Cardio First Angel™ (CFA) device. The primary outcome was sustained return of spontaneous circulation (ROSC), and secondary outcomes were survival to ICU and hospital discharge.

**Results:**

One thousand four hundred fifty-four subjects were randomized; 900 were included. Sustained ROSC was significantly improved in the CFA group (66.7% vs. 42.4%, *P* < 0.001), as was survival to ICU discharge (59.8% vs. 33.6%) and survival to hospital discharge (54% vs. 28.4%, *P* < 0.001). Outcomes were not affected by intra-group comparisons based on intubation status. ROSC, survival to ICU, and hospital discharge were noted to be improved in inter-group comparisons of non-intubated patients, but not intubated ones.

**Conclusion:**

Use of the CFA compression feedback device improved event survival and survival to ICU and hospital discharge.

**Trial registration:**

The study was registered with Clinicaltrials.gov (NCT02845011), registered retrospectively on July 21, 2016.

## Introduction

Effective chest compression remains the cornerstone of successful cardiopulmonary resuscitation (CPR) [1–7]. International guidelines note the critical importance of compression components including position, rate, force, depth, interruptions, recoil, excessive ventilation avoidance, no-flow time, and flow fraction [4–8]. However, observational data suggest that compressions delivered in practice may be suboptimal [9]. Strategies that improve guideline adherence may improve cardiac arrest outcomes. Real-time audiovisual feedback (AVF) and post-event debriefing have been identified as two such strategies [4, 10–12].

Several chest compression feedback devices have been marketed. Those not associated with automated external defibrillators (non-AED) require active chest compression, and most utilize passive decompression. The associated feedback technology ranges in complexity from a simple metronome to electromagnetic sensing [7, 13–16]. Despite a paucity of evidence, the American Heart Association (AHA) and the International Liaison Committee on Resuscitation (ILCOR) have both made cautious recommendations supporting AVF device use [4, 6, 17].

To date, nine non-AED active compression-passive decompression feedback devices have been tested in simulation and clinical environments [7, 13–16, 18–22]. Only one study assessing the use of a hand-held AVF device during in-hospital cardiac arrest (IHCA) has been published [7]. In a study of patients with IHCA (*n* = 80), significant improvements in return of spontaneous circulation (ROSC) rates, guideline adherence, CPR quality, and decreased rib (not sternum) fracture rates were observed for patients receiving compressions using the Cardio First Angel™ (CFA) device [7]. In the current study, we aim to determine if use of the CFA compression feedback device will improve rates of sustained ROSC, and survival to intensive care unit (ICU) and hospital discharge for patients with IHCA.

## Methods

### Study design and settings

**Fig. 1:**
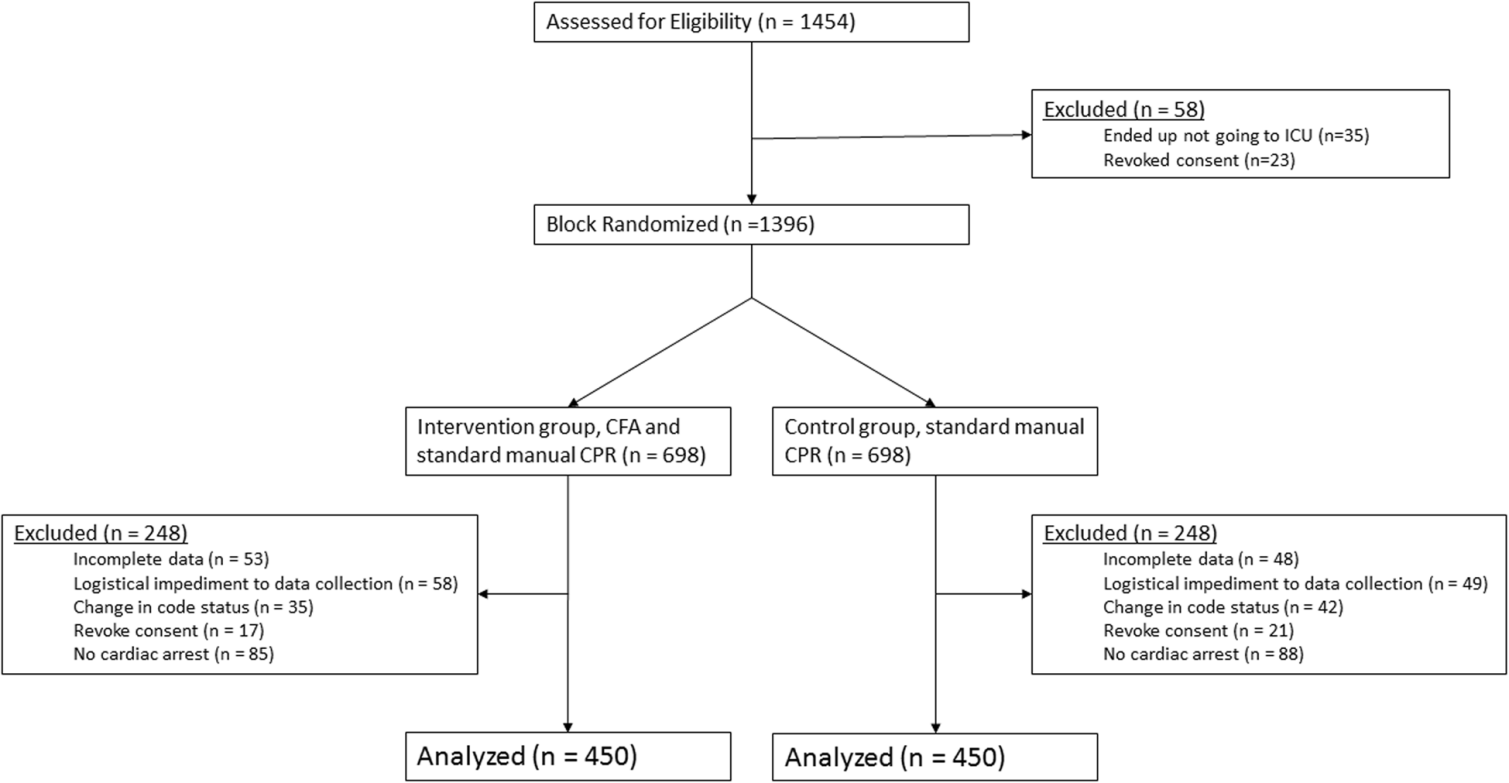
Flow diagram of patient enrollment

This was a prospective, randomized, controlled, parallel study of patients undergoing resuscitation with chest compressions for IHCA in the mixed medical-surgical ICUs of eight academic tertiary care hospitals in Iran from January 1, 2015, to September 15, 2015. All parts of the study were reviewed according to the Consolidated Standards for Reporting Trials (CONSORT) statement (Fig. 1) [23]. The trial was registered with Clinicaltrials.gov (identifier NCT02845011). The protocol is available for review upon reasonable request. Crossover was not allowed. Patients were blinded to randomization group. The healthcare provider was not blind during the resuscitation, as it was considered unethical to employ a sham device. The data analyzer was blinded to group randomization and was not present during resuscitation.

Block randomization (groups of 4) was performed using a random number list generated by Random Allocation Software© (RAS; Informer Technologies, Inc., Madrid, Spain; Fig. 1). Numbers were placed into sequential containers that were kept in a secure location until allocation consignment. To ensure blinding, the allocation sequence was kept by a different investigator than the one enrolling participants. A third investigator was responsible for patient follow-up and assessments. Enrollment and randomization occurred in the emergency department from available admitted ICU patients on a convenience basis. Only patients admitted to the ICU through the emergency department were eligible for inclusion. Patients suffering decompensation in clinical status on the floor or intermediate care units requiring transfer to the ICU were not eligible for study inclusion. Patients consented to enrollment in a study on cardiac arrest treatment should that event occur during the ICU stay. A container was kept at the foot of the bed that either contained the CFA device (intervention) or a weight but no device (control). Upon resuscitation, the container was opened, and providers proceeded with the resuscitation accordingly. There were no important changes to methods after trial commencement. The study ended because it achieved the necessary sample size.

### Patient population

The pre-defined inclusion criteria were as follows: (1) age ≥ 18 years, (2) admitted to the ICU from the emergency department (ED), (3) resuscitation status (full code), and (4) informed consent. Patients were excluded from inclusion if pregnant. Subjects excluded from the final analysis were as follows: (1) any out-of-hospital cardiac arrest or ED cardiac arrest prior to study enrollment, (2) change in code status to anything not *full code*, (3) revoked consent, or for (4) lost or incomplete data due to logistical impediment to data collection. Consent decisions were accepted from either the patient or appropriate legal guardian or surrogate. Decisions to cease resuscitation efforts were made by the team leader in accordance with the European Resuscitation Council and AHA Guidelines for Resuscitation Ethics and included (1) asystole for > 20 min in the absence of a reversible cause [e.g., hypothermia at time of arrest, cardiac tamponade, tension pneumothorax, distributive shock from anaphylaxis, and chemical intoxication/overdose (e.g., opiate)], (2) > 30 min of resuscitation with no occurrence of ventricular fibrillation (VF) or ventricular tachycardia (VT) at any point (initial or subsequent rhythm), (3) injury not compatible with life, (4) severity of comorbidities, and (5) normothermia [24, 25]. For those patients in persistent pulseless VF or VT not responsive to CPR, defibrillation, and medications, the determination to cease resuscitation efforts was made by the resuscitation team leader based on the clinical variables including the following: witnessed versus unwitnessed arrest, time to CPR initiation, comorbid disease, and pre-arrest state.

### Intervention

All arrests were classified as *witnessed* and *monitored* as they occurred in the ICU. Resuscitation teams were comprised of an intensivist, three to five ICU nurses, and a respiratory therapist. Resuscitation was in accordance with standard guidelines, including chest compressions performed by experienced ICU nurses, defibrillation (both automated and conventional available), indicated medications (epinephrine, vasopressin, atropine, amiodarone, sodium bicarbonate, calcium chloride, magnesium sulfate), and ventilation with or without endotracheal intubation [8, 26]. Defibrillation technique for all participants was unchanged from the standard baseline technique. Prior to study deployment, all ICU nurses at approved study sites received standardized CPR training in accordance with published guidelines and training on CFA device use [8, 26]. During resuscitation, patients in the control group received CPR in accordance to published guidelines, whereas patients in the intervention group received compressions with the aid of the CFA feedback device. Measurement of invasive hemodynamics was outside the scope of this study.

### Cardio First Angel™ device

**Fig. 2:**
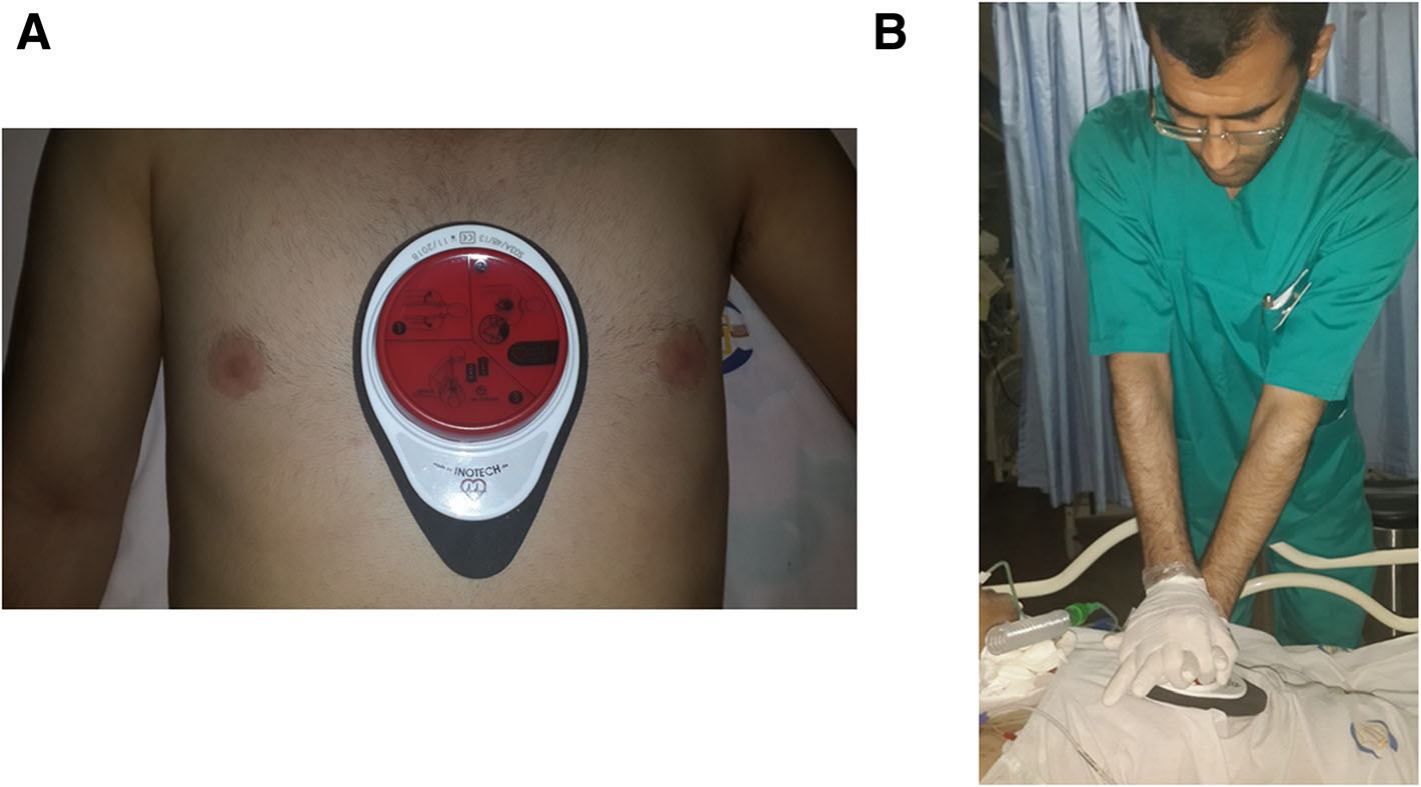
Proper deployment (**a**) and rescuer position (**b**) while using the Cardio First Angel™ device

The Cardio First Angel™ is a handheld device consisting of three components. The rescuer side has a red palm-sized push button with a pictogram illustrating proper use (Fig. 2) [7]. The center unit is composed of a stable plastic base containing an arrangement of springs, and the patient side consists of liquid-absorbent polyurethane foam [7]. Application of 400 ± 30 N of force results in an audible *click* alerting the rescuer to cease compression, and an additional *click* on decompression alerts the rescuer to resume compression [7].

### Data collection

Utstein variables were published after data collection; however, all available Utstein variables are reported [27]. The primary outcome was sustained ROSC (> 30 min). Secondary outcomes were survival to ICU and hospital discharge. Recorded data included age, sex, invasive mechanical ventilation status upon code onset, ICU and hospital length of stay (LOS), diagnoses, presence of known osteoporosis, initial cardiac rhythm, defibrillation, and administered drugs. Trial outcomes were not changed after trial commencement. Data for invasive arterial monitoring and wave form capnography were not routinely available for patients and is not reported. The assessment for multiple-organ dysfunction included (1) respiratory [ratio of partial pressure arterial oxygen and fraction of inspired oxygen (PaO_2_/FiO_2_), minute ventilation (MV)], (2) hematology [platelets, white blood cells], (3) liver [bilirubin, prothrombin time], (4) cardiovascular [mean arterial pressure, systolic blood pressure, heart rate, vasopressor requirement], (5) CNS [Glasgow coma score], and (6) renal [creatinine, blood urea nitrogen, urine output].

### Availability of data and materials

All relevant data are within the paper and its supporting information files. De-identified individual subject data may be available from the corresponding author on reasonable request.

### Sample size and data analysis

The sample size was based upon the survival and morbidity data reported from the pilot study [7] and was performed using STATA® 14 (StataCorp LLC, College Station, TX, USA). Assuming an alpha of 0.05 and a power of 0.9, the necessary sample size per group was 413. Accounting for anticipated 10% attrition, the final sample size needed was 450 per group.

All analyses were performed using SPSS 22.0 (SPSS Inc., Chicago, IL, USA). Descriptive statistics were calculated for all variables. Normality was assessed via Shapiro–Wilk test. Normally distributed continuous variables were compared using the *t* test, with non-normally distributed variables compared via Mann–Whitney *U* test. Categorical variables were compared via chi-square and Fisher’s exact test, as appropriate. An alpha of 0.05 was significant. Multivariate binary logistic regression was performed with backward elimination (Wald) method. No interim analysis was planned or conducted.

## Results

During the 9-month study period, the average hospital admission and expiration rates were 21,940 and 657 (3%), respectively. The average ICU admission and mortality rates per institution were 1693 (13,544 total) and 199 (1592 total, 12%), respectively. Of 1454 subjects approached for enrollment, 1396 were randomized, 554 were excluded (58 pre-randomization; 496 post-randomization), and 900 were included (Fig. 1). Patient demographics were similar between groups (Table 1), as were admission diagnoses (*p* = 0.62): (CFA vs. control) trauma (5% vs. 4%), neurological (20% vs. 17%), renal (24% vs. 24%), cancer (26% vs. 24.7%), respiratory (21% vs. 23%), and abdominal infection (4% vs. 6%). Time from ICU admission to cardiopulmonary arrest (10.5 ± 5.7 days vs. 10.3 ± 4.2 days, *P* = 0.38) and initial recorded rhythm were similar between groups (Table 2). Total electricity dose administered for defibrillation was similar between groups (627 J vs. 623 J, *P* = 0.93), as was first shock success rate (10.9% vs. 12.7%; *P* = 0.47; Table 2) and resuscitation drug and therapy administration (Table 3). Sustained ROSC rates were improved in the intervention group (66.7% vs. 42.4%, *P* < 0.001; Table 2), as was survival to ICU discharge (54% vs. 28.4%, *P* < 0.001) and survival to hospital discharge (54% vs. 28%, *P* < 0.001). Intra- and inter-group comparisons for the first and second half of the study are shown in Table 4. The CFA group outperformed controls in achieving ROSC and ICU survival in each subgroup comparison (Table 4). No intra-group difference was noted for ROSC or ICU survival in the CFA group over time; however, both improved over time in the control group. Intra- and inter-group comparisons for patients intubated (before or during CPR) versus those not intubated is shown in Table 5. No difference in ROSC or survival to discharge was noted within either group when comparing intubated to non-intubated patients. Similarly, no difference in ROSC or survival to ICU or hospital discharge was observed between groups for intubated patients. A significant difference, however, was noted for ROSC (66.8% vs. 42.8%, *P* < 0.0001), survival to ICU discharge (60.3% vs. 33.3%, *P* < 0.0001), and survival to hospital discharge (28.3% vs. 54.1%, *P* < 0.0001) between the intervention and control groups for non-intubated patients.

**Table 1:** Summary statistics and the results of the tests for comparing groups for demographic variables

Variable	Total (*n* = 900)		Intervention (*n* = 450)		Control (*n* = 450)		Significance
	Mean ± SD	*N* (%)	Mean ± SD	*N* (%)	Mean ± SD	*N* (%)	*P* value
Age	57.42 (5.81)		57.26 (5.44)		57.57 (6.16)		0.72^a^
ICU length of stay (days)	25.82 (10.88)		26.95 (10.82)		24.69 (10.84)		< 0.001^a^
Nurse ICU experience (years)	21.47 (4.70)		21.48 (5.13)		21.46 (4.24)		0.27^a^
Sex, female		546 (60.7%)		274 (60.9%)		272 (60.4%)	0.89^b^
Intubated *prior* to CPR event, Yes		368 (40.9%)		176 (39.1%)		192 (42.7%)	0.28 ^b^
Intubated *during* CPR event, Yes				34 (7.6%)		36 (8%)	0.83 ^b^
Multi-organ dysfunction, Yes		438 (48.7%)		210 (46.7%)		228 (50.7%)	0.23 ^b^

^a^Mann–Whitney *U* test

^b^Chi-square test

**Table 2:** Summary of resuscitation variables and outcomes

Variable	Intervention		Control		Significance
	Mean ± SD	Frequency *n* = 450 (%)	Mean ± SD	Frequency *n* = 450 (%)	*P* value
CPR duration (min)	41.51 (6.67)		42.54 (6.57)		0.025^a^
Initial cardiac rhythm					0.236^b^
Asystole		185 (41.1)		183 (40.7)	
Ventricular tachycardia		38 (8.4)		33 (7.3)	
Ventricular fibrillation		42 (9.3)		61 (13.6)	
Pulseless electrical activity and bradyarrhythmia		185 (41.1)		173 (38.4)	
First shock success rate		49 (10.9)		57 (12.7)	0.469^b^
Return of spontaneous circulation		300 (66.7%)		191 (42.4%)	< 0.001^a^
Survival status upon ICU discharge					< 0.001^b^
Alive		269 (59.8)		151 (33.3)	
Dead		181 (40.2)		299 (66.4)	
Survival status upon hospital discharge					< 0.001^b^
Alive		243 (54)		128 (28.4)	
Dead		207 (46)		322 (71.6)	

^a^Mann–Whitney *U* test

^b^Chi-square test

**Table 3:** Comparison of resuscitation treatments

Treatment administered				Treatment dose		
Agent	CFA *n* (%)	Control *n* (%)	*p* value^a^	CFA Median (IQR) Mean	Control Median (IQR) Mean	*P* value^b^
Electricity (Joules)	205 (46%)	211 (47%)	0.69	600 (400–800) 623.7	600 (400–800) 623.7	0.93
Epinephrine (mg)	450 (100%)	450 (100%)	1.0	5 (4–6) 4.95	5 (4–6) 5	0.52
Vasopressin	222 (49%)	213 (47%)	0.55	40 (40–40) 40	40 (40–40) 40	1.0
Atropine (mg)	110 (24%)	103 (23%)	0.58	1 (0.5–1) 0.78	1 (0.5–1) 0.77	0.60
Lidocaine (mg)	145 (32)	146 (32%)	0.94	200 (160–200) 176.8	200 (150–200) 172.3	0.32
Amiodarone (mg)	185 (41%)	195 (43%)	0.50	450 (300–450) 386.8	450 (300–450) 390.8	0.60
Sodium bicarbonate (mEq)	146 (32%)	155 (34%)	0.52	89.2 (66.9–133.8) 99.4	89.2 (66.9–133.8) 105.3	0.30

^a^Chi-square test

^b^Mann–Whitney *U* test

*CFA* Cardio First Angel™, *NS* not significant, *mg* milligrams, *g* grams, *mEq* milli-equivalents

**Table 4:** Intra- and inter-group comparisons from the first vs. second halves of the study period

Groups	ICU survival		Return of spontaneous circulation	
	Frequency (%)		Frequency (%)	
	Alive	Death	Yes	No
Cardio First Angel™ 1st half	126 (56)	99 (44)	144 (64)	81 (36)
Cardio First Angel™ 2nd half	143 (63.6)	82 (36.4)	156 (69.3)	69 (30.7)
Control 1st half	90 (40)	135 (60)	116 (51.6)	109 (48.4)
Control 2nd half	61 (27.1)	164 (72.9)	75 (33.3)	150 (66.7)
Significance level	*P* < 0.0001^a^		*P* < 0.0001^a^	
	*P* = 0.103^b^		*P* = 0.231^b^	
	*P* = 0.001^c^		*P* = 0.008^c^	
	*P* < 0.0001^d^		*P* < 0.0001^d^	
	*P* < 0.0001^e^		*P* < 0.0001^e^	
	*P* < 0.0001^f^		*P* < 0.0001^f^	
	*P* = 0.004^g^		*P* < 0.0001^g^	

^a^Chi-square test comparing the variables in all groups

^b^Chi-square test comparing intra-group comparison of Cardio First Angel™ 1st vs. 2nd half

^c^Chi-square test comparing inter-group comparison for 1st half of study

^d^Chi-square test for inter-group comparison for Cardio First Angel™ 1st half to control 2nd half

^e^Chi-square test for inter-group comparison for Cardio First Angel™ 2nd half to control 1st half

^f^Chi-square test comparing inter-group comparison for 2nd half of study

^g^Chi-square test comparing intra-group comparison of controls 1st vs. 2nd half

**Table 5:** Intra- and inter-group comparison of the effect of intubation status on return of spontaneous circulation and survival to hospital discharge

Airway status	ROSC		Inter-group *P* value ^a^	Survival to ICU discharge		Inter-group *P* value ^a^	Survival to hospital discharge		Inter-group *P* value ^a^
	Control (*n* = 191)	Intervention (*n* = 300)		Control (*n* = 151)	Intervention (*n* = 269)		Control (*n* = 128)	Intervention (*n* = 243)	
Intubated *n* (%)	14 (38.9%)	22 (64.7%)	0.31	13 (36.1%)	18 (52.9%)	0.157	11 (30.6%)	18 (52.9%)	0.057
Non-intubated *n* (%)	177 (42.8%)	278 (66.8%)	< 0.0001	138 (33.3%)	251 (60.3%)	< 0.0001	117 (28.3)	225 (54.1%)	< 0.0001
Intra-group *P* value	0.53	0.80		0.74	0.4		0.77	0.897	

*ROSC* return of spontaneous circulation

^a^Chi-square test

Five variables correlated with sustained ROSC on multivariate analysis including the following: no osteoporosis, no MOD, age < 65 years, initial rhythm of asystole, or ventricular fibrillation (compared to pulseless electrical activity; Table 6). Neither sex, CPR duration, time-of-day (shift), nor admission diagnosis grouping correlated with sustained ROSC. No patients were withdrawn for study-associated harms.

**Table 6:** Variables associated with sustained return of spontaneous circulation as identified through multivariate logistic regression

Step 1a	*β*	S.E.	Wald	df	Significance	Exp (*β*)	95% CI for EXP (*β*)	
							Lower	Upper
Osteoporosis	.325	.136	5.723	1	.017	1.384	1.061	1.807
MOD	−.275	.136	4.101	1	.043	.759	.582	.991
Age < 65 years	.327	.142	5.311	1	.021	1.387	1.050	1.832
Rhythm			7.979	3	.046			
Asystole	.723	.321	5.064	1	.024	2.060	1.098	3.865
VT	.202	.225	.805	1	.370	1.224	.787	1.904
VF	.457	.227	4.062	1	.044	1.579	1.013	2.463
PEA and bradyarrhythmia	−.371	.238	2.441	1	.118	.690		

*MOD* multiple organ dysfunction, *VT* ventricular tachycardia, *VF* ventricular fibrillation, *PEA* pulseless electrical activity

## Discussion

A large gap exists between current knowledge of CPR quality and its optimal implementation, contributing to preventable deaths attributable to cardiac arrest [28]. As such, quality chest compression remains a focal point of international guidelines [5, 6], with important components including compression rate, force, depth, interruptions, allowing adequate recoil, and avoiding excessive ventilation [2, 3, 29, 30]. One would expect strategies that improve guideline adherence to improve patient outcomes following IHCA. Real-time AVF is one such strategy identified by the AHA and ILCOR as an area needing further investigation [6, 9, 11, 12, 25]. In the 2015 *International Consensus on Cardiopulmonary Resuscitation and Emergency Cardiovascular Care Science with Treatment Recommendations*, feedback device use was recommended to provide directive feedback on compression rate, depth, release, and hand position during training (weak recommendation, low-quality evidence). Furthermore, in the absence of AVF devices, tonal guidance (examples include music or metronome) during training is recommended to improve compression rate (weak recommendation, low-quality evidence) [4].

Real-time AVF may be provided by a range of devices, from basic metronomes to devices using accelerometers, springs, or electromagnetic sensing. A recent meta-analysis reported significant improvements in CPR quality, but not ROSC, with AED-associated AVF device use, but no available clinical studies for non-AED devices [31]. One subsequent clinical trial (*n* = 80) using the CFA device reported improved ROSC rates when compared to standard manual compressions [7]. This was followed by simulation studies using laypersons that showed improved hand position, mean compression depth, rate, and compression fraction with the CFA device [20, 21]. Several other non-AED compression feedback devices have shown promising results in simulation studies but lack clinical testing [7, 15, 16, 18, 22, 32].

This prospective randomized controlled study assessed the effect of using a non-AED hand-held compression AVF device on clinical outcomes in patients with IHCA. The overall rate of ICU mortality (12%) was similar to that reported in studies of adults in western ICU [33]. It is known that patient mortality may vary greatly between institutions and countries [34]. As shown in Table 4, the overall ROSC rate (55%) in this study was similar to prior reports from Iran (20%–56%) [7, 35–37], Brazil (71%) [38], USA (43%–52%) [39, 40], Europe (54%–73%) [41–44], Turkey (49%) [45], Japan (65%) [46], and Taiwan (58%–67%) [47, 48] and greater than China (36%) [49], Hong Kong (38%) [50], and Australia and New Zealand (46%) [51]. Although the overall discharge survival rate was higher than previous cohorts, this was due to improved survival in the experimental group. When looking only at the control group, the discharge survival rate was similar to many prior published cohorts (Table 7) [7, 39–43, 46, 51].

**Table 7:** Comparison of international in-hospital cardiac arrest cohorts

Region	Reference	Sample size	Witnessed (%)^a^	Location: ICU, CCU, OR (%)	Mean age (years)^a^	Female (%)^a^	VF/VT (%)^a^	ROSC (%)^a^	Survival to hospital discharge (%) ^a^
Current study									
Total		900	100	100	57	61	18	55	42
CFA		450	100	100	57	61	18	67	54
Control		450	100	100	58	60	21	42	28
ANZ ^b^	[51]	1733	80	NR	NR	36	31	46	25
Austria	[44]	1041	NR	96	64	37	39	NR	36^c^
Brazil	[38]	89	100	100	59	48	15	71	9
China	[49]	2712	NR	7	62	32	57	36	9
Hong Kong	[50]	431	60	17	71-74^d^	38	6	38	5
India	[56]	105	100	100	51	25	5	38	1
Iran	[7]								
Total		80	100	100	61	61	58	54	NR
CFA		40	100	100	60	55	58	73	
Control		40	100	100	63	66	58	35	
Iran	[36]	80	100	NR	67	52	NR	56	
Iran	[37]	600	NR	26	NR	42	NR	45	3
Iran	[35]	206	NR	31	54	41	NR	20	5
Ireland	[41]	741	75	42	66	32	NR	56	18
Ireland	[42]	63	87	11	74	37	NR	73	27
Japan	[46]	491	77	25	71	38	28	65	28^e^
Norway	[43]	302	85	1	70	36	NR	54	25
Taiwan	[47]	110	NR	NR	67	29	14	67	18
Taiwan	[48]	702	50	21	67	39	88	58	6
Turkey	[45]	134	NR	32	67	51	18	49	13
UAE	[66]	685	NR	46	57	34	9	38	8
USA	[54]	14,720	86	51	68	43	25	44	17
USA	[39]	16,245	88	NR	NR	NR	NR	52	23
USA	[59]	25,006	72	37	NR	42	17	50	15
USA	[53]	84,625	100	100	66	42	21	43	17
USA	[60]	471,962	NR	NR	NR	48	NR	NR	18
USA	[40]	235,959	NR	NR	51	42	NR	NR	30

*IHCA* in-hospital cardiac arrest, *ICU* intensive care unit, *CCU* cardiac care unit, *OR* operating room, *VF* ventricular fibrillation, *VT* ventricular tachycardia, *CFA* Cardio First Angel™, *NR* not reported, *ANZ* Australia and New Zealand, *UAE* United Arab Emirates, *USA* United States of America

^a^Numbers rounded to nearest whole number

^b^Meta-analysis of studies from Australia and New Zealand

^c^Duration of survival not specified. Additionally, survival only reported for those with good neurological outcome defined as Glasgow–Pittsburgh cerebral performance categories (CPC) 1 and 2. CPC 1 indicates good capability, CPC 2 indicates slight disability, CPC 3 indicates severe disability, CPC 4 indicates coma or vegetative state, CPC 5 indicates cerebral death. CPC score of 1 or 2 was considered favorable, while a score of 3, 4, or 5 indicated an unfavorable functional neurological outcome

^d^Only range reported

^e^30-day survival. Survival to hospital discharge not reported

Other factors including sex, age, race, and code status may have played a role as well. In modern ICUs, circulatory failure events are often expected and due to gradual deterioration. Often, the goals of treatment are modified resulting in a change in code-status from *Full code* to a *Limited* or *No-Code*/*Do Not Resuscitate* status. Such patients were excluded from this study, resulting in a patient population at higher risk for cardiac arrest (but still salvageable).

Moreover, female sex and lower age have been associated with improved odds of IHCA survival, whereas black race has been associated with decreased survival [40, 52, 53]. Although the sex distribution in this study is consistent with Iran’s national census data, the proportion of females in our study was higher than other published cohorts [38, 40, 42, 44, 50, 53, 54]. Additionally, the mean age in our cohort was slightly lower than that in some prior reports (Table 7) [35, 42, 44, 45, 47–50, 54–56]. Furthermore, the rate of VF/VT may affect outcomes [53], and although the incidence in published reports of IHCA has been quite variable, the 18% noted in our cohort falls mid-spectrum [38, 44–50, 54, 56]. Rates of anti-arrhythmic administration (e.g., Amiodarone) were in keeping with other cohorts [54]. In this study, VF/VT rates were lower than in the pilot study [7], suggesting that factors other than arrhythmia type are driving improvements in ROSC and survival rates. Indeed, a trend toward improved ROSC and survival rates has been observed in IHCA patients, regardless of initial rhythm [53].

As it pertains to resuscitation outcomes over time, we performed both intra- and inter-group comparisons for the first and second halves of the study. ROSC and survival to hospital discharge were consistently observed in the intervention versus control group. This is thought to reflect CFA device use. Moreover, higher rates of ROSC and survival to hospital discharge were observed within the intervention group in the second half of the study period relative to the first. This may be related to greater familiarity and comfort with the CFA feedback device. However, the reasons for improved ROSC and survival to hospital discharge in the control group for the first relative to the second half of the study period remain unclear and necessitate further investigation targeted on this question as the study was not designed for this purpose.

It remains unknown whether endotracheal intubation offers ROSC or survival benefits over bag-valve-mask ventilation or supraglottic airway placement during resuscitation for IHCA. Small non-randomized studies have suggested a benefit [45, 48, 56, 57], whereas others have suggested the absence of need for endotracheal intubation as being associated with improved survival [58]. However, data from a large administrative database study (25,006 cardiopulmonary arrest events) found that early invasive airway insertion was not associated with improved ROSC rates, and only slightly better odds of 24 h survival (adjusted OR 0.94, 0.89–0.99) [59]. Moreover, data from both non-randomized clinical studies [47] and large administrative database [60] studies have reported that patients who were already intubated or had received mechanical ventilation before resuscitation had reduced ROSC and survival. We performed both intra- and inter-group comparisons to further assess the impact of intubation on resuscitation outcome. We found no difference in ROSC, or survival to ICU discharge, or survival to hospital discharge for intra-group comparisons based on intubation status. Furthermore, differences in these outcomes were noted only for inter-group comparisons of non-intubated patients, but not intubated ones. The etiology for this is thought to be due to improved CPR quality in the intervention group, but further investigation focused on this finding are needed to better clarify the matter.

Work environment may also play a role in IHCA survival [61–64]. It has been estimated that an increase by one full-time registered nurse per patient ICU day reduces IHCA relative risk by 28% [65]. Shift time (day, eve, night) does not seem to be a significant factor in IHCA survival in this and other studies [53]. Lastly, the use of therapeutic hypothermia after IHCA varies across studies, and in Iran, it is not common.

### Limitations

This study did not enroll patients with primary cardiac conditions; such patients were admitted to the cardiac ICU. This study was not designed to follow neurologic outcomes, as such, data regarding functional outcome is not available. Unfortunately, the study was not designed to capture data on compression rate, depth, chest recoil, no flow time, or flow fraction. In future studies we will plan to incorporate some (or all) of these variables. Moreover, invasive arterial monitoring and wave form capnography were not routinely available at the time of the study and is not reported.

A significant limitation of compression feedback device studies is the inability to blind the clinical providers. Blinding the subject, the investigator, and the data analysts is easy and was done in this case. But to blind the clinical provider, one would need to either (1) use a sham device or (2) not allow the clinician to “see” the patient being resuscitated during device application and “hide” the device during compression pauses. Sham device use was deemed to be unethical, and the latter impractical. Furthermore, one criticism of the current non-AED compression feedback devices is that they do not account for complex changes that occur during CPR. Like other AVF devices, CFA accounts for neither complex changes in chest wall compliance and elasticity, nor the compressibility of the surface the patient is lying on (e.g., mattress). Lastly, future investigations would benefit from optimization of the methodology to detect morbidity including sternum and rib fractures.

Additionally, assessments for chest wall morbidity (rib or sternum fractures) were not uniform (X-ray, CT scan, autopsy) precluding extrapolation of conclusions. This was in part due to funding and resource limitations. Future investigations should improve upon these limitations.

## Conclusion

This is the first large-scale clinical trial comparing chest compressions using a hand-held non-AED compression AVF device to standard manual chest compressions during IHCA. Use of the CFA device improved sustained ROSC, ICU survival, and survival to hospital discharge. This study is the first of its kind to show that use of an inexpensive compression AVF device may improve patient outcomes from IHCA. This may have tangible effects for both wealthy and resource-limited institutions. Further prospective clinical investigation comparing CFA to other commercially available AVF devices is needed to clarify the performance characteristics and potential benefit of using these devices.

## Abbreviations

AC-PD: Active compression-passive decompression
AED: Automated external defibrillators
AHA: American Heart Association
AVF: Audiovisual feedback
CFA: Cardio First Angel™
CPR: Cardiopulmonary resuscitation
ED: Emergency department
ICU: Intensive care unit
IHCA: In-hospital cardiac arrest
ILCOR: International Liaison Committee on Resuscitation
LOS: Length of stay
ROSC: Return of spontaneous circulation
VF: Ventricular fibrillation
VT: Ventricular tachycardia
